# CD47 Promotes Age-Associated Deterioration in Angiogenesis, Blood Flow and Glucose Homeostasis

**DOI:** 10.3390/cells9071695

**Published:** 2020-07-15

**Authors:** Kedar Ghimire, Yao Li, Takuto Chiba, Sohel M. Julovi, Jennifer Li, Mark A. Ross, Adam C. Straub, Philip J. O’Connell, Curzio Rüegg, Patrick J. Pagano, Jeffrey S. Isenberg, Natasha M. Rogers

**Affiliations:** 1 Centre for Transplant and Renal Research, Westmead Institute for Medical Research, University of Sydney, 176 Hawkesbury Rd, Sydney 2145, NSW, Australia; sohel.julovi@sydney.edu.au (S.M.J.); jennifer.li1@sydney.edu.au (J.L.); philip.oconnell@sydney.edu.au (P.J.O.); 2 Heart, Lung, Blood and Vascular Medicine Institute, University of Pittsburgh, BST Starzl Tower, 200 Lothrop Street, Pittsburgh, PA 15261, USA; yal102@pitt.edu (Y.L.); chibat@upmc.edu (T.C.); astraub@pitt.edu (A.C.S.); pagano@pitt.edu (P.J.P.); 3 Department of Pharmacology & Chemical Biology, University of Pittsburgh, BST Starzl Tower, 200 Lothrop Street, Pittsburgh, PA 15261, USA; 4 Center for Biologic Imaging, University of Pittsburgh School of Medicine, BST, 200 Lothrop Street, Pittsburgh, PA 15261, USA; mross@pitt.edu; 5 Department of Oncology, Microbiology and Immunology, Faculty of Sciences and Medicine, University of Fribourg, Chemin du Musée 18, PER 17, 1700 Fribourg, Switzerland; curzio.ruegg@unifr.ch; 6 Department of Medicine, University of Pittsburgh, BST Starzl Tower, 200 Lothrop Street, Pittsburgh, PA 15261, USA

**Keywords:** aging, angiogenesis, CD47, thrombospondin-1, glucose homeostasis, metabolism, self-renewal, endothelial cells, blood flow

## Abstract

The aged population is currently at its highest level in human history and is expected to increase further in the coming years. In humans, aging is accompanied by impaired angiogenesis, diminished blood flow and altered metabolism, among others. A cellular mechanism that impinges upon these manifestations of aging can be a suitable target for therapeutic intervention. Here we identify cell surface receptor CD47 as a novel age-sensitive driver of vascular and metabolic dysfunction. With the natural aging process, CD47 and its ligand thrombospondin-1 were increased, concurrent with a reduction of self-renewal transcription factors OCT4, SOX2, KLF4 and cMYC (OSKM) in arteries from aged wild-type mice and older human subjects compared to younger controls. These perturbations were prevented in arteries from aged CD47-null mice. Arterial endothelial cells isolated from aged wild-type mice displayed cellular exhaustion with decreased proliferation, migration and tube formation compared to cells from aged CD47-null mice. CD47 suppressed ex vivo sprouting, in vivo angiogenesis and skeletal muscle blood flow in aged wild-type mice. Treatment of arteries from older humans with a CD47 blocking antibody mitigated the age-related deterioration in angiogenesis. Finally, aged CD47-null mice were resistant to age- and diet-associated weight gain, glucose intolerance and insulin desensitization. These results indicate that the CD47-mediated signaling maladapts during aging to broadly impair endothelial self-renewal, angiogenesis, perfusion and glucose homeostasis. Our findings provide a strong rationale for therapeutically targeting CD47 to minimize these dysfunctions during aging.

## 1. Introduction

Aging is associated with and driven by vascular dysfunction and metabolic derangements. Vascular aging and metabolic disorder play a key role in determining the health status of the aged population since they represent independent cardiovascular disease (CVD) risk factors [1]. Manifestations of aging include decreased vascularity and blood flow, impaired wound healing, compromised organ and tissue function and in some individuals, dysregulated glucose and lipid homeostasis. The simultaneous occurrence of diabetes, dyslipidemia, obesity and hypertension is termed metabolic syndrome (MetS) [2]. MetS is found in roughly one third of the U.S. population and is a key risk factor for the development of cardiovascular disease and diabetes [3,4]. At the molecular level, aging coincides with diminution in the salutary signaling moieties such as nitric oxide (NO) and vascular endothelial growth factor (VEGF) and decline in the expression of cellular self-renewal factors among others [5].

CD47 (also called the integrin-associated protein, IAP) is an integral membrane protein that was first co-purified with αvβ3 integrin and is expressed ubiquitously [6]. We previously showed that CD47 binds to thrombospondin-1 (TSP1) to inhibit the beneficial effects of NO [7] and VEGF [8], and drives tissue injury caused by hypoxia, ischemia, reperfusion, radiation and chemotherapy [6]. Skin from aged wild-type (WT) mice displayed increased levels of TSP1 and aged TSP1-null mice were protected from tissue ischemia [9] while CD47 antagonists enabled cellular self-renewal and reprogramming by overcoming negative regulation of c-Myc and other stem cell transcription factors in young cells [10]. However, the role of CD47 in promoting aging through vascular and metabolic imbalance was unknown.

We report that with natural aging, CD47 and TSP1 increase in expression in arteries of aged WT mice and older human subjects, promoting deterioration of multiple restorative and homeostatic mechanisms. Age-related induction of TSP1 and CD47 is accompanied by decreased levels of the crucial self-renewal transcription factors OCT4, SOX2, KLF4 and cMYC (abbreviated OSKM), concurrent with diminished angiogenic capacity. In addition, CD47-null endothelial cells (ECs) from aged animals showed enhanced migration, proliferation and tube formation compared to cells from aged WT controls. Further, CD47 blockade improved the angiogenic response of arteries from older human subjects. Aged CD47-null mice were protected from decrease in angiogenesis, blood flow, high fat diet (HFD)-induced weight gain, glucose intolerance and insulin resistance. Taken together, these findings indicate that CD47 signaling globally promotes aging by suppressing angiogenesis, and escalating vasculopathy and metabolic imbalance.

## 2. Materials and Methods

### 2.1. Animals

All animal studies were performed under protocols approved by the University of Pittsburgh Institutional Animal Care and Use Committee in accordance with NIH guidelines or under protocols approved by the Western Sydney Local Health District Animal Ethics Committee (#5128). Male 3-month old mice C57BL/6 wild-type (CD47^+/+^) and CD47-null mice (strain B6.129S7-Cd47^tm1Fpl/J^) were purchased from Jackson Laboratory (Bar Harbor, ME) and maintained for at least 18 months before being used as aged mice. Mice were fed ad libitum with either a standard chow diet (8% calories from fat; Gordon’s Specialty Stockfeeds, Yanderra, Australia) or a high-fat diet containing lard/sucrose (45% calories from fat, based on rodent diet D12451; Research Diets, New Brunswick, NJ, USA). As described, some of the aged mice received a standard HFD for a total of 16 weeks, starting at 14 months of age.

### 2.2. Reagents

Antibodies used were cMyc (#5605, 1:1000), KLF4 (#4038, 1:1000), Sirt1 (#8469, 1:1000), β-Actin (13E5, #4970, 1:4000), β-Actin (8H10D10, #3700, 1:4000) and total eNOS (#32027, 1:1000) from Cell Signaling (Danvers, MA, USA). Phosphorylated eNOS (p-eNOS) detecting serine 1177 (ab215717, 1:500), anti-glutathione peroxidase-1 antibody (ab108427, 1:1000), SPRED1 antibody [M23-P2G3] (ab64740, 1:1000) and anti-Ki67 antibody (ab15580, 1:300) were from Abcam (Cambridge, MA, USA). CD47 MIAP301 (sc-12731, 1:500) and CD47 B6H12 (sc-12730, 1:500) were from Santa Cruz Biotech (Santa Cruz, CA, USA). Matrigel^®^ Growth Factor Reduced (GFR, Product Number 354230) was from Corning, Inc. (Corning, NY, USA). Endothelial cell growth media (Catalog #: CC-3156) was from Lonza (Basel, Switzerland). VEGF Recombinant Human Protein (#PHC9393) was from ThermoFisher Scientific (Waltham, MA, USA) and reconstituted in PBS according to the manufacturer’s instructions. Dispase^®^ II was from Sigma-Aldrich (St. Louis, MI, USA). Mouse FibrOut™ 11 and Human FibrOut™ custom prepared for Blood Vessels and Endothelial Tissues were from Chi Scientific Inc. (Maynard, MA, USA).

### 2.3. Human Mesenteric Arteries

Fresh small bowel specimens from healthy young and old individuals were obtained from the International Institute for the Advancement of Medicine (IIAM, Edison, NJ, USA; https://www.iiam.org/). Distal 5th-order systemic mesenteric arteries were gently harvested from the small bowel specimens under magnification and employing sterile technique. Strict criteria for IIAM for the exclusion of specimens from diseased individuals ensured that arterial samples varied simply by the age of the donor. The arterial segments all appeared grossly normal and the tissue sections did not show any obvious pathology.

### 2.4. mRNA Isolation and Quantitative Reverse-Transcription PCR

Total RNA was extracted using Qiagen RNeasy^®^ Mini Kits (Qiagen, Hilden, Germany) with on-column DNase digestion. RNA was quantified using a Nanodrop 8000 spectrophotometer (Thermofisher, Waltham, MA, USA) and reverse-transcribed using Superscript III First Strand Synthesis SuperMix (Invitrogen, Carlsbad, CA, USA). cDNA was amplified using Platinum^®^ PCR SuperMix-UDG (Invitrogen) with gene-specific TaqMan primers and probed on the ABI Prism 7900HT Sequence Detection System (Applied Biosystems, Foster City, CA, USA) according to the manufacturer’s instructions. Thermal cycling conditions were 50 °C for 2 min, 95 °C for 2 min, followed by 40 cycles of 95 °C for 15 s and 60 °C for 1 min. Data were analyzed using the ΔΔCt method with expression normalized to the housekeeping gene.

### 2.5. Endothelial Cell (EC) Isolation and Culture

Murine arterial ECs were isolated employing a published protocol [11] with several modifications. Murine thoracic aortas from 18 months old mice were excised and divided into 8–10 square sections using microdissection scissors and implanted on growth factor-reduced Matrigel (Corning, Corning, NY, USA) with the lumen-side faced down. On day 4 after plating, the aortic segments were gently removed from the matrix without disrupting the growing ECs. ECs were allowed to proliferate on the matrix for a further 2–3 days, after which Dispase was used to digest the Matrigel. Freed ECs were plated on 0.1% gelatin-coated 6 cm culture dishes. To specifically select for ECs, CD31-coated magnetic beads (CellBiologics, Chicago, IL, USA) were added to cell suspensions and the non-EC fraction removed. Aged ECs proved fragile and ≥10 murine aortas were needed to isolate sufficient cells to maintain viability in a single culture dish and allow for further experiments.

### 2.6. Protein Extraction and Western Blotting

SDS-PAGE was performed as described previously [12]. In brief, cells were washed twice with ice-cold DPBS and lysed in RIPA lysis buffer (Cell Signaling Technology, Danvers, MA, USA) supplemented with 1 × protease inhibitors cocktail (Sigma, St Louis, MO, USA) and 1 × phosphatase inhibitors cocktail (Roche Applied Science, Hercules, CA, USA). The samples were then centrifuged for 5 min at 10,000× *g*. Protein concentration was determined by BCA (bicinchoninic acid assay) protein assay (Thermo Fisher, Waltham, MA, USA). 20 μg of protein was denatured using Laemmli SDS buffer, heated to 95 °C for 5 min, and subjected to SDS-polyacrylamide gel electrophoresis. ECs were pooled together from ≥10 murine aortas for each group/lane and samples were loaded in three wells. When proteins were at a similar molecular weight, parallel blots were employed to overcome the ineffectiveness of the restriping and reprobing process.

### 2.7. Immunofluorescence

Immunofluorescence staining was performed as described [13] with minor modifications. In short, endothelial cells were seeded onto gelatin-coated 13-mm glass coverslips and incubated overnight. At 70% confluence, media was removed, and cells were washed with PBS, fixed with paraformaldehyde (4%) and permeabilized with Triton X at 0.5% in PBS for 10 min. Cells were then washed with PBS and blocked in 5% bovine serum albumin in PBS for 1 h at room temperature (RT), then incubated with the indicated primary antibodies overnight at 4 °C in a humidified chamber. Alexa Fluor 488 or 568 secondary antibodies were added for 1 h at RT. DAPI (Sigma-Aldrich) was used to stain cell nuclei. Cells were mounted in Prolong Gold (Invitrogen) and fluorescent images were captured with a Nikon Eclipse E 800 microscope.

### 2.8. In Vivo Matrigel Plug Assay and Immunohistochemistry

Mice were injected subcutaneously near the supraspinal midline with 0.5 mL of growth factor reduced Matrigel (Corning, Inc., Corning, NY, USA). After 14 days, Matrigel plugs were removed, fixed with 10% buffered paraformaldehyde, and embedded in paraffin. After tissue fixation and cutting, sections were stained with the anti-CD31 antibody and Masson’s trichrome. Immunohistochemical staining was performed as described [13]. CD31-positive vessel-like structures per high-powered field (100 μm × 100 μm; ×20 magnification) were counted from five Matrigel plugs and averaged for each genotype. The blood vessel quantification per field of view was semiquantitative and vessels containing or devoid of RBCs were counted for both genotypes. Quantification of staining was performed using ImageJ program (NIH, Bethesda, Rockville, MD, USA). Hemoglobin content in the plugs was measured using Drabkin’s reagent (Sigma-Aldrich, #D5941).

### 2.9. Tube Formation Assay

Endothelial tube formation assays were performed following a published protocol [14]. 6 × 10^4^ WT and CD47-null aortic ECs were seeded onto the growth-factor reduced Matrigel in 24 well plates. Cells were pooled from ≥10 animals from each group. Tube formation was monitored on an hourly basis and images taken at 5× magnification. Tube formation parameters (total tube length, branching length and number of nodes) were calculated using the Angiogenesis Analyzer Program for ImageJ (NIH, Bethesda, Rockville, MD, USA).

### 2.10. Arterial Ring Assay

Arterial ring assays were performed following a published protocol [15]. In brief, murine thoracic aortas and human distal mesenteric arteries were dissected and extraneous fat, tissue and branching vessels removed and cut into 1 mm wide rings. Rings were placed in Opti-MEM for 4 h before embedding into the growth-factor reduced Matrigel with cell media. In some instances, rings were incubated with a CD47 blocking (clone B6H12) or IgG control antibody, with VEGF or with both. Sprouts were counted daily in several replicate wells and images acquired. Mouse FibrOut™ 11 and Human FibrOut™ custom prepared for blood vessels and endothelial tissues was added to the cell media following the manufacturers’ protocol to suppress fibroblast growth.

### 2.11. Scratch Wound Healing Assay

Murine aortic ECs (1 × 10^3^ cells/well) were isolated, pooled from ≥10 aortas from WT and CD47-null mice and seeded onto Lab-Tek chambered cover glass with complete media (5% FBS) and cultured until confluent. A wound was made by scraping the cell monolayer with a sterile 10 uL pipette tip. The culture medium was changed immediately to remove detached cells and debris. Cultures were incubated for 24 h in a live microscope chamber and images were taken at various timepoints. Open wound area measurements were performed using the TScratch program and ImageJ (NIH, Bethesda, Rockville, MD, USA). Hoechst stain was used to stain the DNA and the 10× objective was used to track the wound inside a 2-well LabTek dish. Images were captured every 30 min for 24 h. Three stage positions per condition were acquired at each time point. Distance (as pixels) moved by individual cell fronts on both sides of the scratch were obtained and plotted on one side. ROIs (region of interest) were made from the nuclei and the distance of migration by cells was calculated using Nikon Elements software available with the Nikon Live Cell 218E microscope and obtained values were divided by the time taken to close the wound. Measurements were taken at 100 separate points in 3 separate wells for each condition.

### 2.12. Cell Proliferation Assay

ECs (1 × 10^3^ cells/well) were isolated and pooled together from ≥10 aortas from WT and CD47-null mice and seeded onto a 96-well tissue culture plate and incubated for 24 and 48 h. After incubation, wells were washed to remove debris. Cell proliferation was determined using the CyQUANT^®^ Cell Proliferation Assay (Life Technologies, Grand Island, NY, USA). The comparative proliferative capacity of aged WT and CD47-null endothelial cells was calculated as the number of cells (based on total DNA content) by measuring fluorescence at 520 nm and comparing it to the known cell number intensity as a reference. The assay was done in quadruplicate and performed twice. JuLi^TM^Br live microscopy was utilized to measure confluence of ECs in tissue culture dish for CD47 re-expression experiments. For transient knockdown of CD47, the mouse CD47 siRNA with sequence 5′-GGAAUGACCUCUUUCACCA-3′ and the control siRNA with sequence 5′-AATTCTCCGAACGTGTCACGT-3′ synthesized by Ambion Inc. were used. After knockdown, tumor necrosis factor-alpha (TNF-α, 50 ng/mL, R&D) was added to ECs to upregulate CD47; 24 h prior to placing the dishes inside the JuLi^TM^Br machine.

### 2.13. Laser Doppler Blood Flow Analysis

Hind limb blood flow was assessed in young and aged WT and CD47-null mice. Mice were anesthetized with 2.5% isoflurane and placed in a supine position on a heating pad. Core temperature was maintained at 37 °C and monitored continuously by a rectal probe thermometer. Prior to scanning, skin was removed to expose the underlying vascular bed and muscle. Real-time blood flow was measured using laser Doppler imaging (Moor LDI-2λ; Moor Instruments, Devon, UK) and results are expressed at basal flux values obtained from the instrument.

### 2.14. Glucose (GTT) and Insulin (ITT) Tolerance Test

For the GTT, mice were fasted for 6 h in the morning. At time 0, a tail cut was made, a blood sample taken and fasting blood glucose level (BGL) measured using a Stat Strip Glucometer (NovaBiomedical, Runcorn, UK). A glucose bolus (2 g/kg body weight) was injected intraperitoneally and BGL measured at 5, 10, 15, 20, 30, 45, 60 and 120 min post- injection. For the ITT, mice were fasted for 3 h and fasting BGL measured. Then, an insulin bolus (0.75-1 U/kg body weight) was injected intraperitoneally and BGL measured at 5, 10, 20, 30, 45, 60, 120 and 180 min post-injection. The area under the curve (AUC) of the glucose measurements was calculated.

### 2.15. Statistical Analysis

Data are presented as the mean ± SEM (unless otherwise indicated) of the results from at least 3 independent cell cultures, at least 6–8 animals per group or 3–6 human samples per group. Comparisons were made using an unpaired Student’s *t* test, one-way or 2-way ANOVA according to the data type, followed by Tukey’s test for multiple comparisons. A *p* < 0.05 was considered statistically significant.

## 3. Results

### 3.1. Expression of CD47 Is Increased and Self-Renewal Transcription Factors Decreased in Aged Arteries

**Figure 1:**
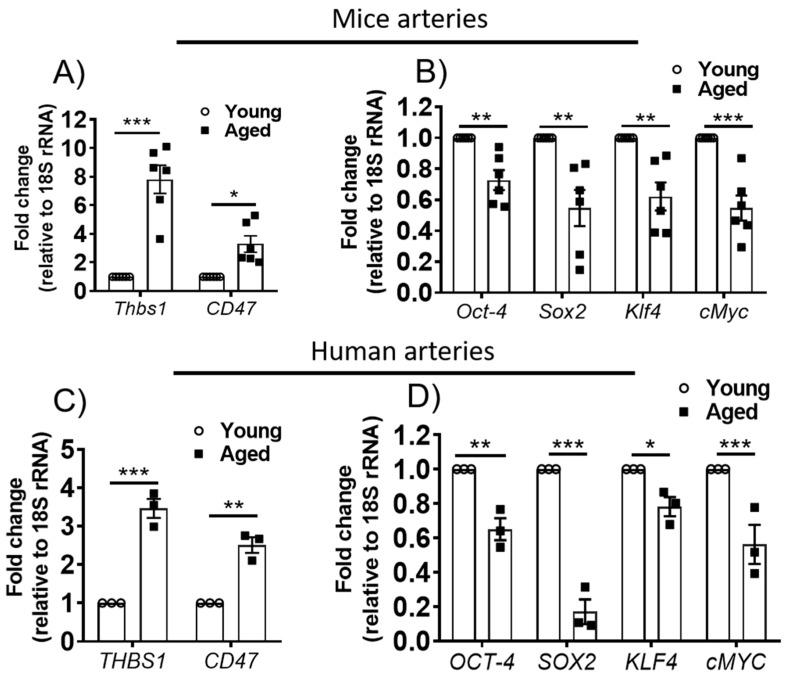
TSP1 and CD47 are increased and self-renewal transcription factors decreased in arteries from aged animals and older individuals. Gene expression profiling by q-PCR of *Thbs1* and *Cd47* (**A**) and self-renewal factors *OCT4, SOX2, KLF4* and *cMYC* (**B**) in aortas from young (3-month-old) and aged (18-month-old) mice and in distal 5^th^-order mesenteric arteries from young (<33 years) and old (>60 years) healthy human subjects (**C**,**D**). Error bars represent the mean ± SEM, samples in triplicate/mouse, 3–5 mice/group. Samples in triplicate/individual, 3 subjects/group. Data normalized to the 18srRNA gene. An unpaired *t*-test was performed with * *p* < 0.05, ** *p* < 0.01, *** *p* < 001.

It is not known what role aging plays in arterial CD47 expression. qPCR analysis of aortas from aged (18-months-old) wild-type (WT) mice showed that both the ligand *Thbs1* and the receptor *Cd47* transcript levels increased with age compared to younger (3-months-old) controls (Figure 1A). With aging, the capacity for self-renewal is lost [16,17]. Several transcription factors are essential for self-renewal including OCT4, SOX2, KLF4 and cMYC (OSKM) [18]. The effect of age on the arterial expression of these key self-renewal factors was unknown. Interestingly, mRNA levels of the OSKM factors were decreased in arteries from aged WT mice compared to vessels from young animals (Figure 1B). To explore the translational relevance of this observation, mesenteric arteries from young (<33 years) and old (>60 years) otherwise healthy human subjects were obtained. The demographic characteristics of this cohort are presented in the supplemental files (Supplementary Figure S1A). Arterial segments that looked grossly normal were utilized for the experiments. Consistent with findings in arteries from aged mice, *Thbs1* and *Cd47* mRNA levels were elevated (Figure 1C), albeit not to similar levels as found in mice. OSKM mRNA levels also decreased in arteries from older compared to younger individuals, with *Sox2* levels decreased almost 5-fold (Figure 1D).

### 3.2. Age-Associated Induction of TSP1 Is Attenuated and OSKM Sustained in the Absence of CD47

Our previous findings show that TSP1 is increased in the pulmonary vasculature of older individuals [19,20]. Furthermore, TSP1, via CD47, suppresses self-renewal transcription factors in cell culture [10]. However, it was unclear if CD47 was required for age-associated induction of vascular TSP1 and alterations in OSKM expression. In arteries from aged WT mice, both *Thbs1* and *Cd47* transcript levels increased significantly (Supplementary Figure S1B,C) while OSKM expression (Supplementary Figure S1D–G) decreased compared to vessels from younger controls. Interestingly, induction of arterial TSP1 was prevented in arteries from aged CD47-null mice compared to aged WT animals (Supplementary Figure S1B,C). OSKM mRNA levels were generally maintained in arteries from aged CD47-null mice except *cMyc*. While *cMyc* mRNA levels were reduced, expression was still greater compared to vessels from aged WT controls (Supplementary Figure S1D–G).

### 3.3. Age-Associated Decrease in Arterial Endothelial Cell Proliferation is Abrogated in the Absence of CD47

**Figure 2:**
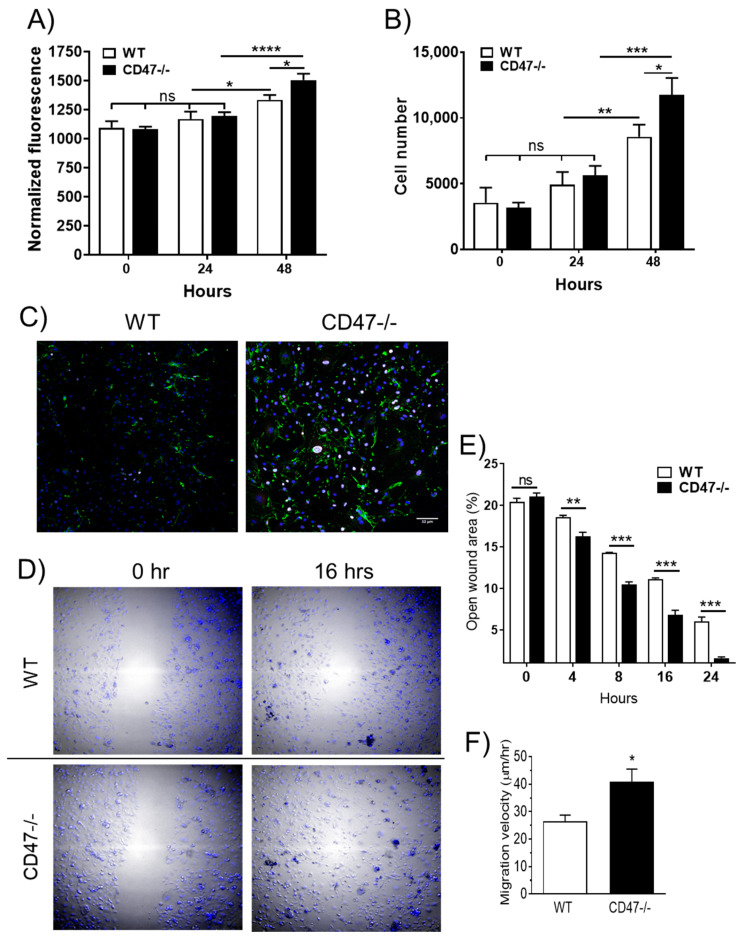
Age-associated decreases in arterial endothelial cell (EC) proliferation and migration are abrogated in the absence of CD47. Aortic ECs from aortas from aged male wild-type (WT) and CD47-null mice were characterized for DNA content using CyQUANT^®^ fluorescence (**A**) and cell count (**B**). Ki67 immunofluorescence (in white) in arterial ECs of arteries from aged WT and CD47-null mice, von Willebrand factor in green and DAPI in blue (**C**). Representative images of ECs after scratch wounding at 0 and 16 h (**D**). Quantification of the open wound area in respective images (**E**). Quantification of average cell migration velocity (**F**). Scale bar: 32 μm. Error bars represent the mean ± SEM, samples in triplicate wells. An unpaired *t*-test was performed with * *p* < 0.05, ** *p* < 0.01, *** *p* < 0.001, ns: non-significant.

Endothelial cell (EC) proliferation is necessary for angiogenesis and is lost with aging [21]. TSP1 and a CD47-specific binding domain of TSP1, inhibited NO-mediated proliferation of ECs from young mice [7,22]. Additionally, pulmonary ECs from young CD47-null mice exhibited increased proliferation compared to cells from young WT mice [10]. However, the effect of CD47 signaling on the proliferation of aged mouse aortic endothelial cells (MAECs) is not known. Arterial ECs isolated from aged mice (both WT and CD47-null) proliferated minimally and were not different in their proliferative capacity during the first 24 h (Figure 2A, B). In contrast, by 48 h, wild-type ECs lagged significantly in proliferation compared to CD47-null cells (Figure 2A,B), and this correlated with increased expression of the cell proliferation marker Ki67 [23] in CD47-null cells (Figure 2C).

To confirm that these findings were CD47-specific, we conducted CD47 rescue experiments in CD47-depleted ECs. Previous work showed that acute re-expression of CD47, using a human CD47 plasmid, in CD47-null lung endothelial cells promoted apoptosis [10]. Intracellular upregulation of CD47 has recently been described where TNF-NFKB1 signaling directly regulates CD47 expression by interacting with a constituent enhancer located within a CD47-associated super-enhancer region [24]. CD47 expression in aged wild-type ECs was transiently silenced first using siRNA to achieve a partial loss-of-function, and then cell were stimulated with TNF-α for 24 h to physiologically restore CD47. The attempt to upregulate CD47 in aged wild-type ECs in this manner also led to significant cell death, possibly secondary to the overall fragility of aged endothelial cells. To overcome this, we used ECs from 12 month old mice, which tolerated CD47 upregulation better than aged cells (Supplementary Figure S2A). Using JuLi^TM^Br live microscopy, a time-course of cell confluency between wild-type, CD47-depleted and CD47-re-expressed ECs was measured. Consistent with aged EC proliferation data, we noted that CD47 knock down (CD47 KD) led to increased proliferation of 12-month aged ECs. CD47 KD ECs reached 90% confluency within 12 h whereas wild-type ECs only achieved around 65% confluency in the same period (Supplementary Figure S2B). More importantly, we observed that increasing CD47 levels in CD47-depleted cells could rescue its wild-type behavior. But increasing CD47 levels in CD47-depleted cells resulted in their their enhanced proliferative capacity to decrease below that of wild-type ECs (Supplementary Figure S2B).

### 3.4. CD47 Contributes to Age-Associated Decrease in Endothelial Cell Migration

Angiogenesis relies on the migration of ECs in addition to proliferation [25]. We examined the migratory capacity of aged WT and CD47-null ECs in scratch wound assays. Arterial ECs obtained from aged WT mice resurfaced scratch wounds slower than cells from aged CD47-null mice (Figure 2D,E), and unlike proliferation, these differences manifested within 8 h of wounding. By 16-h post-injury, CD47-null ECs had nearly covered the area of the scratch wound (Figure 2D and Supplementary Figure S2C). Consistent with this, aged CD47-null ECs demonstrated a greater average migration velocity compared to WT cells (Figure 2F).

### 3.5. CD47 Limits Tube Formation by Endothelial Cells from Aged Mice

**Figure 3:**
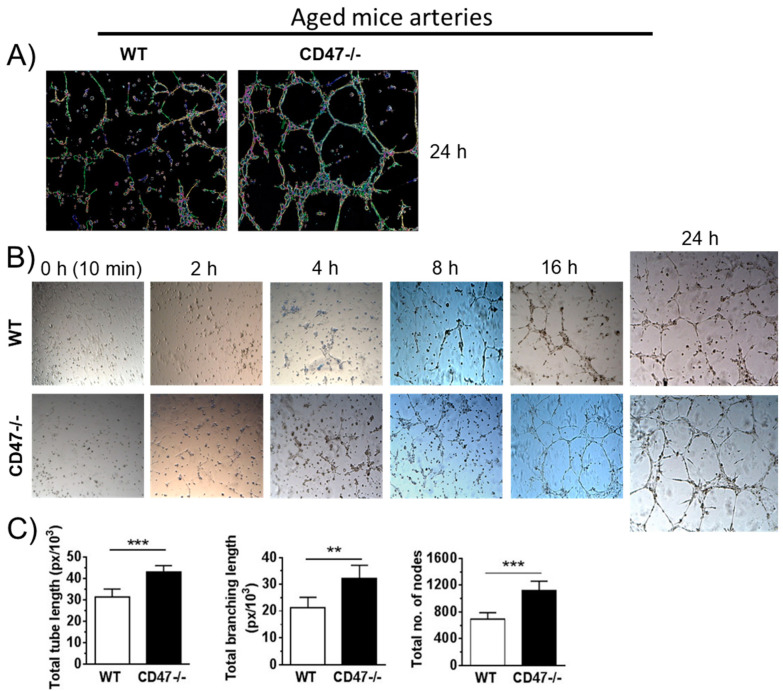
CD47 limits tube formation by endothelial cells from aged mice. Representative images (**A**,**B**) and the quantification (**C**) of total tube length, total branching length and total number of nodes of arterial ECs from aged male wild-type (WT) and CD47-null mice. Error bars represent the mean ± SEM. An unpaired *t*-test was performed with ** *p* < 0.01, *** *p* < 0.001.

ECs form capillary-like structures (tubes) when cultured on 3D extracellular matrix, reflecting a propensity to form the inner lining of blood vessels [14]. This process recapitulates several features of in vivo angiogenesis. Arterial ECs isolated from aged wild-type and CD47-null mice were isolated and cultured for 2 days. Then they were seeded onto growth factor-depleted Matrigel. At 24 h, quantification of tube formation parameters including tube length, branch length and the number of branch points (nodes) was performed (Figure 3A). ECs from aged CD47-null mice were superior in all three angiogenic readouts compared to cells from aged WT animals (Figure 3A–C).

### 3.6. Sprouting Angiogenesis in Aged Human and Murine Arterial Rings Is Hindered by CD47

**Figure 4:**
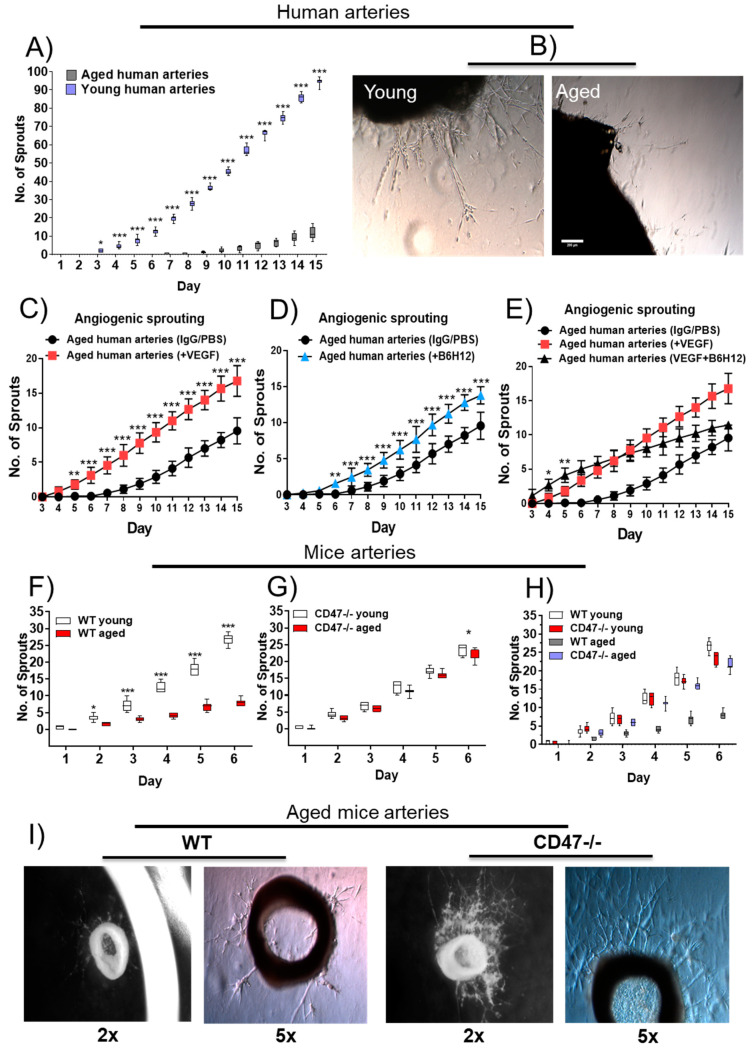
Sprouting angiogenesis in aged human and murine arterial rings is hindered by CD47. Quantification (**A**) and representative images (**B**) of sprouting of distal 5^th^-order human superior mesenteric arteries from old (>60 years) and young (<33 years) individuals. Human arteries from old (>60 years) versus young (<33 years) individuals were cultured in the presence of VEGF (50 ng/mL) (**C**) or a human CD47 blocking antibody (clone B6H12, 2 μg/mL) (**D**) or antibody + VEGF (**E**) and angiogenic sprouting quantified. Quantification of aortic sprouting from 3- and 18-month-old male wild-type (WT) and CD47-null mice (**F**,**G**), and side-by-side comparison (**H**), with representative images of aged mice artery sprouting (**I**). Scale bar: 200 μm. Error bar represents the mean ± SEM, 3 wells/vessel, 3 subjects/group for human arteries and 3 wells/vessel and 3 mice/group. * *p* < 0.05, ** *p* < 0.01, *** *p* < 0.001; 1-way ANOVA, followed by a Tukey’s multiple comparison test.

Unlike mono-cellular assays, the arterial ring assay in Matrigel encompasses a more complex and physiological angiogenic response [15]. Arterial rings from young individuals displayed a faster onset and significantly greater amount of endothelial sprouting compared to arteries from older human subjects (Figure 4A,B). Indeed, angiogenic sprouting took over twice as long to become evident in arterial rings from old compared to young individuals (7 versus 3 days).

To further characterize the response, arterial rings from older subjects were treated with vascular endothelial growth factor (VEGF), a well characterized inducer of angiogenesis [26]. Aged human arterial rings treated with VEGF displayed an accelerated onset and overall greater degree of endothelial cell sprouting compared to control-treated (IgG in PBS) rings (Figure 4C). It was not clear if CD47 signaling played a part in these results, therefore arterial rings from older individuals were treated with a CD47 blocking antibody (clone B6H12). Angiogenic sprouting increased in CD47 antibody-treated rings, similar in levels to rings treated with VEGF, compared to controls (Figure 4B, and Supplementary Figure S3A for side-by-side comparison). Interestingly, treatment of arteries with both VEGF and CD47 antibody shortened the time for the initiation of sprouting but suppressed total sprouting at later time points compared to VEGF alone (Figure 4E). To further confirm the relevance of CD47 in the age-mediated decrease in angiogenic sprouting, sprouting was investigated in arteries from young and aged wild-type (WT) and CD47-null mice. Consistent with findings in human arteries, vessels from aged WT mice had noticeably deficient sprouting compared to vessels from young animals (Figure 4F). In contrast, arterial rings from aged CD47-null mice had cell sprouting comparable to vessels from young mice (Figure 4G,H), which was persistently greater than sprouting from aged WT animals (Figure 4H,I). TSP1 has previously been implicated in aging and inflammation [20,27]. To determine whether the effects of CD47 are cell autonomous or dependent on TSP1 in aged arteries, we monitored angiogenic sprouting in aged WT and CD47-null aortic rings after incubation with exogenous TSP1 (2.2 nM). TSP1 significantly inhibited sprouting from day 4 in aged WT rings compared to controls, but this inhibition tapered off at day 8 (Supplementary Figure 3B). Interestingly, addition of exogenous TSP1 was ineffective in inhibiting the increased sprouting seen in aged CD47-null aortic rings (Supplementary Figure S3C,D for side-by-side comparison). Nonetheless, compared to controls, TSP1-treated rings had slightly lower sprouting on each counted day (Supplementary Figure S3D).

### 3.7. CD47 Suppresses In Vivo Angiogenesis in Aged Mice

**Figure 5:**
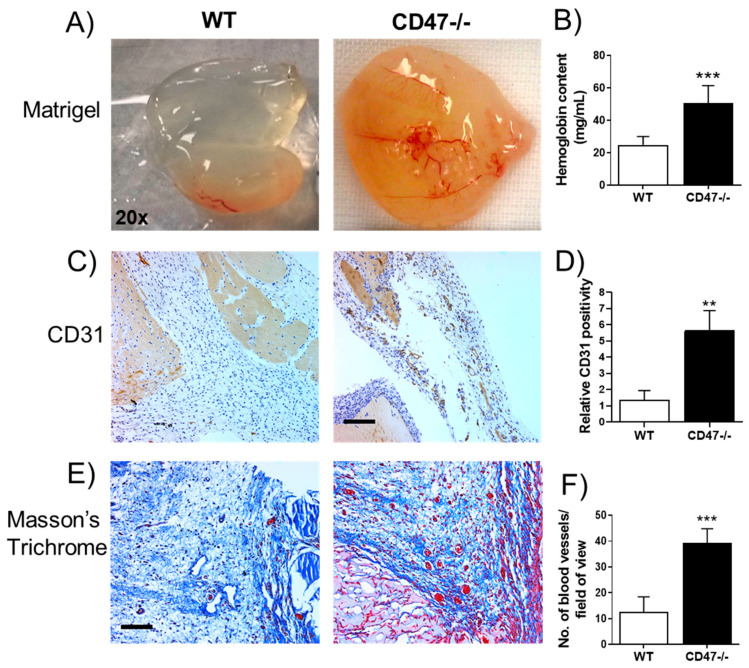
CD47 suppresses in vivo angiogenesis in aged mice. Inspection of angiogenesis (**A**), Matrigel plug hemoglobin content (**B**) and CD31 expression and quantification (**C**,**D**). Quantification of blood vessels in Matrigel plugs obtained from aged CD47-null compared to aged wild-type (WT) mice (**E**,**F**). Representative whole Matrigel plug images (20× magnification), *n* = 6/group. Scale bar: 100 μm. Error bars represent the mean ± SEM. An unpaired *t*-test performed with ** *p* < 0.01, *** *p* < 0.001, ns: non-significant.

The role of CD47 in angiogenesis during old age in vivo is unknown. The Matrigel plug assay is used to assess in vivo angiogenic capacity [28]. Matrigel plugs implanted in aged CD47-null mice showed significantly greater angiogenic activity compared to plugs from aged wild-type (WT) controls (Figure 5A). Increased hemoglobin content (Figure 5B) and endothelial-specific CD31 expression confirmed the presence of blood vessels and endothelial cells (Figure 5C,D). In addition, Masson’s trichrome staining showed a noticeably greater number of RBC-filled blood vessels in plugs from aged CD47-null mice compared to WT controls (Figure 5E,F). Plugs implanted in aged WT mice showed a minimal angiogenic response.

### 3.8. Youthful Blood Flow Is Maintained in Aged CD47-Null Mice

**Figure 6:**
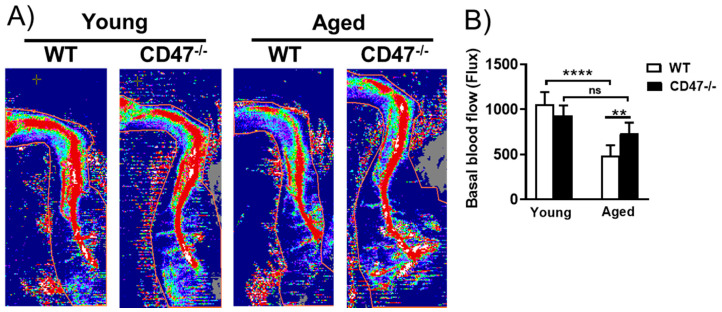
CD47 drives age-related deterioration of hind limb blood flow. Representative laser Doppler images and their quantification (**A**,**B**) of blood flow in the hind limbs of young and aged wild-type (WT) and CD47-null mice. Error bars represent the mean ± SEM. 1-way ANOVA, followed by Tukey’s multiple comparison test for blood flow measurement, 5–6 mice/group. An unpaired *t*-test performed with ** *p* < 0.01, **** *p* < 0.0001, ns: non-significant.

Aging male TSP1-null mice display better hind limb reperfusion following femoral artery ligation compared to wild-type (WT) controls [29]. TSP1 interacts with cell receptors other than CD47 [30] and it is not known whether CD47 impacts baseline blood flow with advancing age. Employing laser Doppler analysis using our established protocol, hind limb blood flow was measured in 3 and 18-month-old animals. Blood flow markedly decreased with age in aged WT mice compared to young ones (Figure 6A,B). Blood flow in young CD47-null mice was slightly lower compared to wild types, but not significantly so. Remarkably, blood flow failed to decrease in aged CD47-null mice compared to young nulls, as well as compared to aged WT animals (Figure 6A,B).

### 3.9. CD47 Limits Angiogenic Gene Expression and Matrix Metalloproteinases in Endothelial Cells from Older Animals

**Figure 7:**
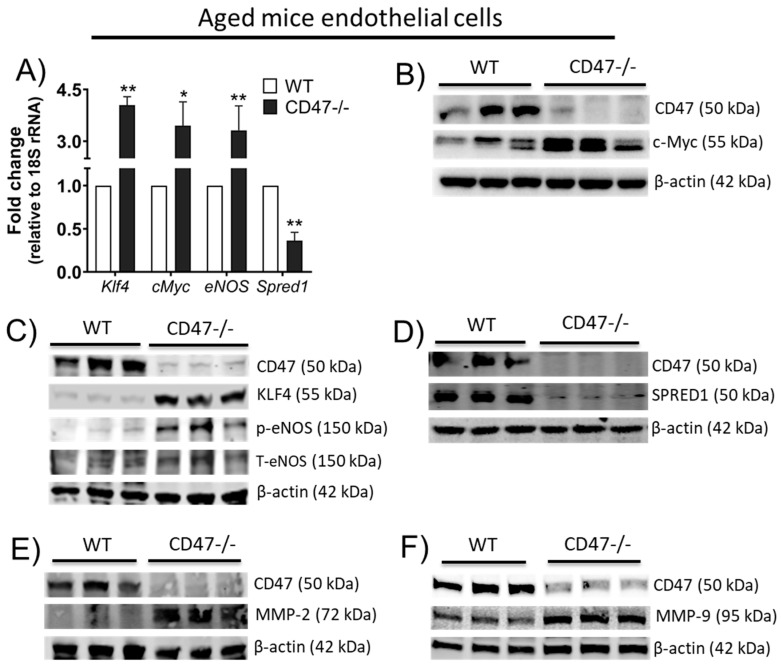
CD47 alters expression of pro- and anti- angiogenic moieties in endothelial cells from aged mice. Arterial EC from aged wild-type (WT) and CD47-null mice were subjected to mRNA (**A**) and protein (**B**–**F**) expression analysis for the indicated molecules. Aortic ECs isolated from aged WT and CD47-null mice (≥10 vessels/group, *n* = 10) were pooled together and samples loaded in wells. Error bars represent the mean ± SEM. An unpaired *t*-test performed with * *p* < 0.05, ** *p* < 0.01.

JinB8 cells (a human T cell line lacking CD47) exhibited increased VEGF receptor levels compared to control cells, while VEGF mRNA and protein levels were increased in young CD47-null mice compared to young wild-type mice [31]. These data suggested that age-associated induction of CD47 might suppress pro-angiogenic signaling moieties. Screening for angiogenic genes revealed that aortic ECs from aged CD47-null mice displayed increased transcript and protein levels of cMyc, KLF4 and phosphorylated (active) eNOS (Figure 7A–C). Interestingly, sprouty-related, EVH1 domain-containing protein 1 (SPRED1), a negative regulator of VEGF signaling [32], was downregulated in aged CD47-null ECs compared to aged WT cells (Figure 7A,E). These results show that CD47 limits a broad range of angiogenic signals. To understand the mechanism of pro-angiogenic bias in CD47-null aged ECs, we examined whether CD47 expression was associated with changes in matrix metalloproteinases (MMPs) levels. MMPs are responsible for the degradation of a number of extracellular matrix (ECM) components, including MMP- 2 and 9 in the basement membrane [33]. Cleavage of the basement membrane is an essential cellular event for angiogenesis [33]. MMP-2 and MMP-9 levels were increased in CD47-null aged ECs (Figure 7E,F).

### 3.10. Age-Related Aspects of MetS Are Mitigated in the Absence of CD47

**Figure 8:**
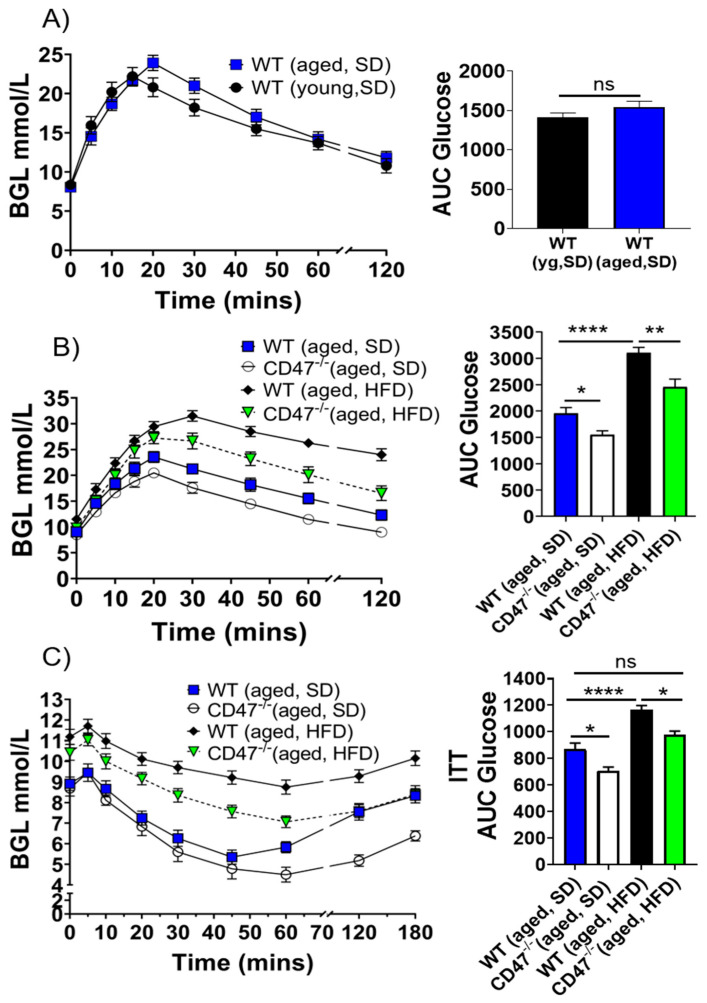
Aspects of age-related metabolic syndrome are attenuated in the absence of CD47. Intraperitoneal glucose clearance test results between young and aged wild-type (WT) mice (**A**), between aged WT and CD47-null mice on standard diet (SD) and high fat diet (HFD) (**B**). Insulin sensitivity test results between aged WT and CD47-null mice on SD and HFD (**C**). Six to eight animals/group. BGL: Blood glucose level. Error bars represent the mean ± SEM. An unpaired *t*-test between two groups and 1-way ANOVA, followed by a Tukey’s multiple comparison test for more than 2 groups. * *p* < 0.05, ** *p* < 0.01, **** *p* < 0.0001, ns: non-significant.

Together with diminished angiogenesis and blood flow, the aging process is associated with glucose intolerance, insulin resistance and obesity, all characteristics of MetS [34]. Metabolic imbalance acts in a feed-forward manner to promote aging and age-associated vasculopathy. The TSP1 promoter is glucose sensitive [35] and cells obtained from young diabetic TSP1-null mice show increased tube formation [36]. Conversely, vascular cells cultured in high glucose display decreased insulin-like growth factor-1 signaling and enhanced CD47 proteolysis [37]. Young (12-week-old) CD47-null mice fed a high fat diet (HFD) weigh less than age-matched HFD-fed wild-type (WT) mice [38]. Together these data suggested that the CD47 signaling axis might impact MetS. Consistent with previous findings [39], glucose-challenged aged WT mice on a standard diet (SD) showed a modest, but not significant impairment in glucose clearance compared to younger counterparts (Figure 8A). However, aged CD47-null mice displayed superior glucose tolerance compared to aged WT animals (Figure 8B). This suggested that CD47 receptor-mediated signaling might promote certain aspects of age-associated MetS. Aged 14-month old WT and CD47-null mice were fed a HFD (45% fat) for 16 weeks and glucose tolerance assessed. Aged CD47-null mice resisted weight gain on a HFD (Supplementary Figure S4) and displayed significantly better glucose clearance compared to aged WT mice (Figure 8B). Loss of glucose tolerance may be due to decreased insulin secretion or sensitivity [40]. Whether CD47 plays any role in glucose homeostasis is not known. SD-fed aged CD47-null mice displayed slightly higher sensitivity to insulin compared to WT mice shortly (0–30 min) after insulin administration (Figure 8C). However, the absence of CD47 significantly extended the duration and magnitude of insulin response in later phases (50–180 min). Under the metabolic stress of a HFD, aged WT mice became insulin resistant, showing minimal physiological response to exogenous hormone, while aged CD47-null mice remained more sensitive to insulin, at both the early and late time points (50–180 min; Figure 8C).

## 4. Discussion

In the vasculature, aging is accompanied by decreased endothelial cell renewal, angiogenesis and blood flow, as well as derangements in metabolism. Herein, we identified a novel role for CD47 in the promotion of age-related perturbations in ECs, including angiogenesis, blood flow and in wole animals, glucose homeostasis. Aging itself was sufficient to upregulate arterial TSP1 and CD47 expression, while simultaneously downregulating essential self-renewal factors genes (OSKM) in rodent and human arteries. These data are in line with reports of increased renal and myocardial TSP1 expression with age [41,42] and extend our previous findings in human pulmonary arteries that TSP1 expression positively correlated with advancing age [20]. Together, our data support the idea that vascular CD47 expression is age-sensitive.

In the absence of CD47, vascular OSKM expression was resistant to age-mediated suppression, suggesting a dominant role for CD47 to limit OSKM in the aging vasculature. The inverse relationship between CD47 and OSKM manifested in reduced angiogenic capacity in vessels from old mice and human subjects. CD47 depletion enhanced proliferation and migration of ECs isolated from aged WT mice while CD47 re-expression curtailed the proliferative advantage seen in null-cells showing that these effects are CD47-specific. Indeed, CD47 re-expression inhibited proliferation of CD47-depleted ECs to levels lower than that of wild-type ECs, which could be due to higher cell death. Additionally, TNF caused CD47 levels to rise in CD47-depleted cells to levels higher than seen in wild-type ECs. The age-related increase in TSP1 expression was not found in arteries from aged CD47-null mice indicating that ligandand receptor tandemly express with age. Interestingly, exogenous TSP1 did not inhibit the higher degree of sprouting achieved by aged CD47-null aortic rings. These data demonstrate that effects of CD47 are cell-autonomous in aging and TSP1-independent mechanisms may contribute to the overall angiogenic effects of CD47.

Arteries from aged CD47-null mice had angiogenic capacity comparable to arteries from young WT animals. Of therapeutic importance, a CD47 blocking antibody moderately improved the angiogenic capacity of arteries from older individuals to levels, similar to but not quite as much as achieved with VEGF. Given the increased expression of CD47 in aged cells, it is possible that the concentration of CD47 antibody employed in the explant assay experiments was insufficient to obtain full target coverage. Interestingly, combined treatment with a CD47 antibody and VEGF accelerated angiogenic sprouting but limited the final degree of sprouting, suggesting possible antagonistic effects under these conditions. These data build on our prior findings that CD47 blockade improves NO-mediated vaso-relaxation in individuals with advanced vasculopathy [19]. 

Hind-limb blood flow also varied between aged WT and CD47-null animals. Whereas WT mice showed a steep drop in blood flow (50%) with age, the CD47-null animals did not. Maintenance of hind-limb blood flow in aged CD47-null mice was likely secondary to multiple factors including the absence of CD47 signaling and the presence of enhanced NO and VEGF signaling. Greater overall vascularity in CD47-null tissues, and variations in autonomic regulation of vascular tone, may have also contributed to these findings. 

Nitric oxide sustains the expression of OCT4 and SOX2 [43] and, in feed-forward signaling, upregulates self-renewal genes in young cells [43]. Thus, changes observed in OSKM and pro-angiogenic genes may, in part, be secondary to the established inhibitory effects of CD47 signaling on NO [44,45]. Regardless, in the absence of CD47-mediated signaling, ECs were resistant to age-mediated loss of angiogenic capacity. To our knowledge, this is the first study to characterize the angiogenic capacity of aortic ECs from 18-month old mice. Parenthetically, aged CD47-null ECs tolerated cell passage better than aged wild-type cells (data not shown) providing additional evidence that with aging, CD47 limits endothelial self-renewal. CD47 was also found upstream of a repertoire of pro-angiogenic and regenerative genes. In aged aortic ECs, previously unknown relationships between CD47 and pro-angiogenic KLF4, cMyc and anti-angiogenic SPRED1 were uncovered. This latter novel finding warrants further study as SPRED1 inhibits angiogenesis through inhibition of microRNA-126 [46]. Increased MMP activity is essential for normal angiogenesis. Here too, CD47 limited MMP expression in aged ECs. These findings provide alternative mechanisms by which CD47 promotes age-related ischemia and vasculopathy. It remains to be seen how the single cell surface receptor CD47 functions to simultaneously regulate multiple angiogenic and self-renewal genes. It alludes to the possibility of CD47 being a major signaling node that modulates angiogenic and self-renewal signals during aging.

Metabolic syndrome (MetS) is a characteristic feature, and known stimulator, of aging. Aged CD47-null mice responded with a more rapid normalization of blood glucose following a glucose bolus compared to wild-type animals. cMYC functions to increase glucose uptake [47]. Increased cMYC in CD47-null animals may contribute to improved glucose tolerance [48]. Glucose and sodium-glucose transporters regulate glucose transit across the cell membrane. The role of CD47 in regulation of these systems is unknown but could also explain changes in blood glucose seen in aged null mice. Additionally, aged CD47-null mice displayed greater insulin sensitivity compared to wild types on SD and, under HFD, maintained this sensitivity for an extended time period. Overall, aged CD47-null mice weighed less than aged wild types, and resisted weight gain following HFD. These findings are relevant as TSP1 may be a clinical marker of impending MetS [49,50] while a lack of CD47 protects young animals from diet-driven weight gain and glucose intolerance [38,51]. Skeletal muscle insulin signaling maintains glucose balance and human skeletal muscle expresses both CD47 and signal regulatory protein 1 (SIRP-α) [52]. CD47 is a ligand of SIRP-α, the latter of which interacts with the insulin receptor [53], suggesting alternative CD47 signaling pathways may contribute to age-related metabolic dysfunction.

This study has certain limitations. First, the findings were based on the analysis of ECs and arteries from male mice. NO signaling is modified by gender [54,55]. Additionally, TSP1 inhibits vaso-relaxation in coronary arteries from aged but not young female rats [56] further alluding to possible gender-specific aspects of TSP1-CD47 signaling. Second, as both TSP1 [57] and CD47 [58] can modulate integrin signaling, our data could also reflect changes in cell adhesion as a cause of modifying angiogenesis. Third, weight changes in wild-type and CD47-null mice may represent differences in energy utilization as we previously reported [59]. Weight differences between mouse strains could also be secondary to variation in food intake or activity level, which were not characterized. Fourth, the Matrigel assay is partially inflammation-dependent and macrophage-derived TSP1 could play a role in inflammation-induced angiogenesis [60]. However, bone marrow transplant studies showed that parenchymal, not the circulating signal TSP1, was also important in limiting certain types of tissue injury [61]. Lastly, interpretation of studies in human arteries may reflect unknown co-morbidities among the organ donors and variations in tissue ischemic intervals.

Aging promotes vasculopathy and metabolic dysregulation while vasculopathy and metabolic dysregulation promote aging. Our findings show that CD47 signaling is upregulated with age in systemic arteries and ECs, concurrent with suppression of key self-renewal genes. A lack of CD47 enhances EC self-renewal, angiogenesis, maintains tissue perfusion and glucose homeostasis, while retarding weight gain, despite increasing age. CD47 blockade improves angiogenic capacity in aged human arteries. Together, these results identify CD47-mediated signaling as an age-sensitive mechanism promoting multiple negative ramifications, such as endothelial exhaustion, hypoperfusion and aspects of MetS. Anti-CD47 therapies may enhance vascular capacity, tissue perfusion and metabolism in the face of advancing age.

## Supplementary Materials

The following are available online at https://www.mdpi.com/2073-4409/9/7/1695/s1, Figure S1: Demographic characteristics of organ donors; Age-associated induction of TSP1 is attenuated, and OSKM sustained, in the absence of CD47. Figure S2: Restoring CD47 in CD47-depleted ECs using TNF-α. Increasing CD47 levels rescues wildtype behavior in aortic ECs from12-month-old CD47-null mice, with representative JuLi™Br images on the right; CD47 delays restoration of endothelial scratch wounds, representative images of endothelial cell mono-layer scratch wounds at 0 and 16 h. Figure S3: Comparison of angiogenic sprouting in human and mouse arteries under various conditions. Figure S4: Absence of CD47 retards HFD-induced weight gain with age.
