# Supplementary material for: CD47 Promotes Age-Associated Deterioration in Angiogenesis, Blood Flow and Glucose Homeostasis
Source: Cells. 2020 Jul 15;9(7):1695. doi: 10.3390/cells9071695 (PMC7407670; doi:10.3390/cells9071695)
Supplement: Supplementary file 1 [file cells-09-01695-s001.pdf]

cells

Supplementary Materials

Supplementary Figure 1

A)

		Demographic Information			
No.	Age (yrs)	Gender	Weight (kg)	Ethnicity	Cause of Death
1	28	M	62	African American	Head Trauma
2	32	F	55	Caucasian	Anoxia
3	21	M	98	Hispanic	Head Trauma
4	61	F	70	Caucasian	Trauma (gunshot)
5	63	M	68	Caucasian	Cerebrovascular Accident
6	72	M	76	Caucasian	Trauma (vehicle accident)

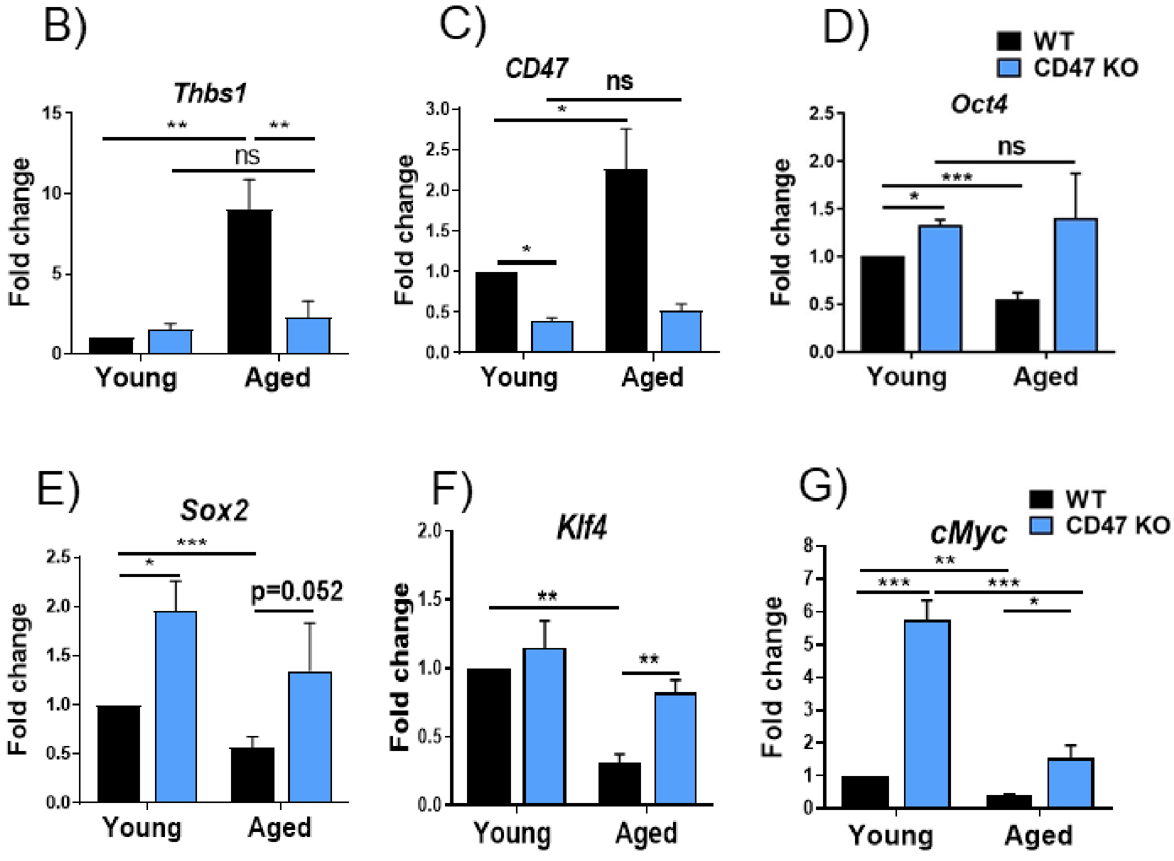

Supplementary Figure 1. Demographic characteristics of organ donors (**A**); Age-associated induction of TSP1 is attenuated, and OSKM sustained, in the absence of CD47 (**B**–**G**). Gene expression profiling by q-PCR of *Thbs1* and *Cd47* (**A** and **B**) and self-renewal factors *OCT4, SOX2, KLF4,* and *cMYC* (**C**–**F**) in aortas from young (3-month-old) and aged (18-month-old) mice. Error bars represent the mean ± SEM, samples in triplicate/mouse; 3-5 mice/group. Data normalized to 18srRNA gene. *$p < 0.05$, **$p <$ 0.01, ***$p <$ 001.

Supplementary Figure 2

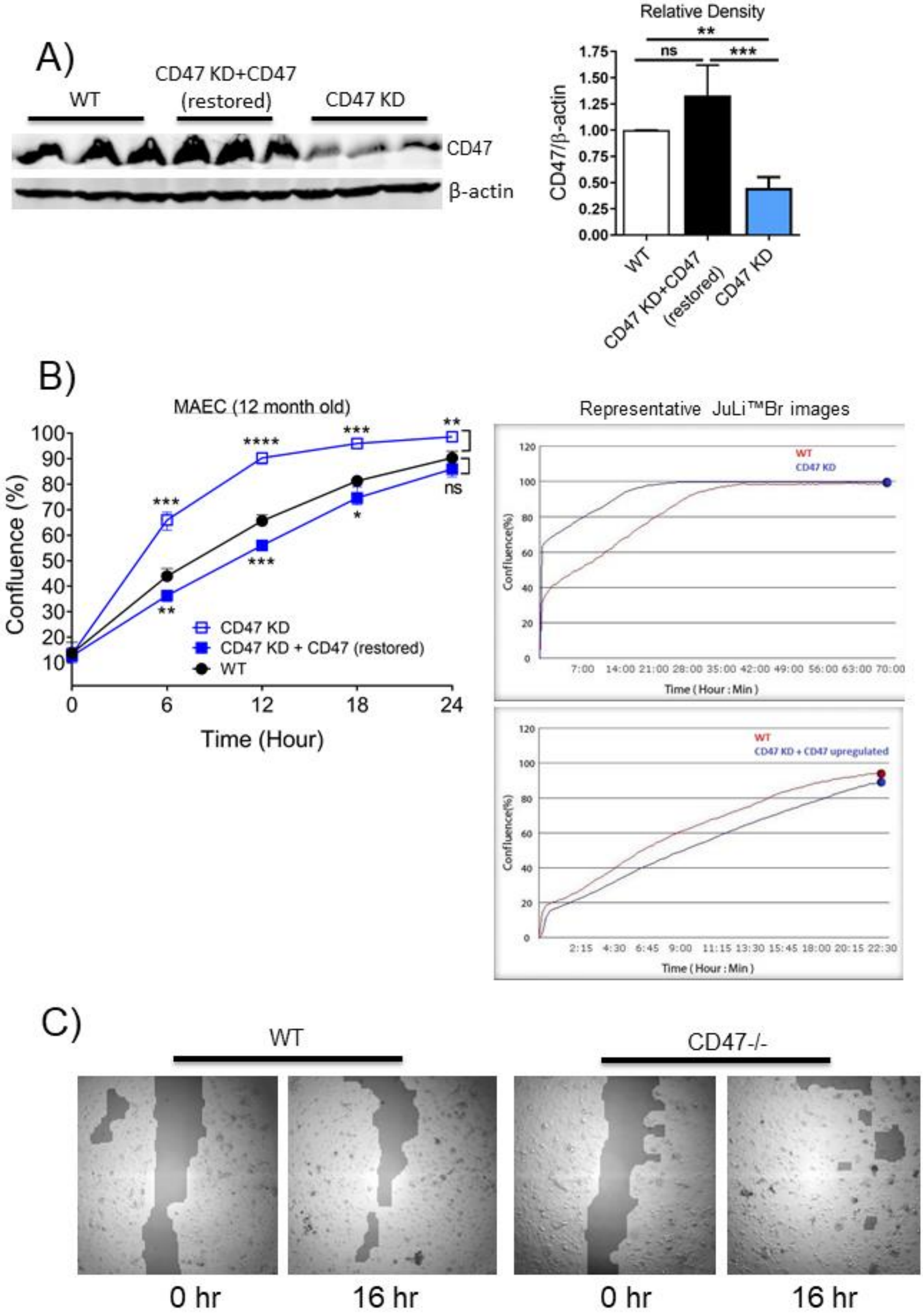

Supplementary Figure 2. Restoring CD47 in CD47-depleted ECs using TNF-α (50 ng/mL),middle lanes (**A**). Increasing CD47 levels rescues wildtype behavior in CD47-depleted 12 month old mouse aortic ECs (MAEC) (**B,** left graph), with representative JuLi™Br images on the right. CD47 delays restoration of endothelial scratch wounds (**C**), with representative images of endothelial cell monolayer scratch wounds at 0 and 16 h.

Supplementary Figure 3

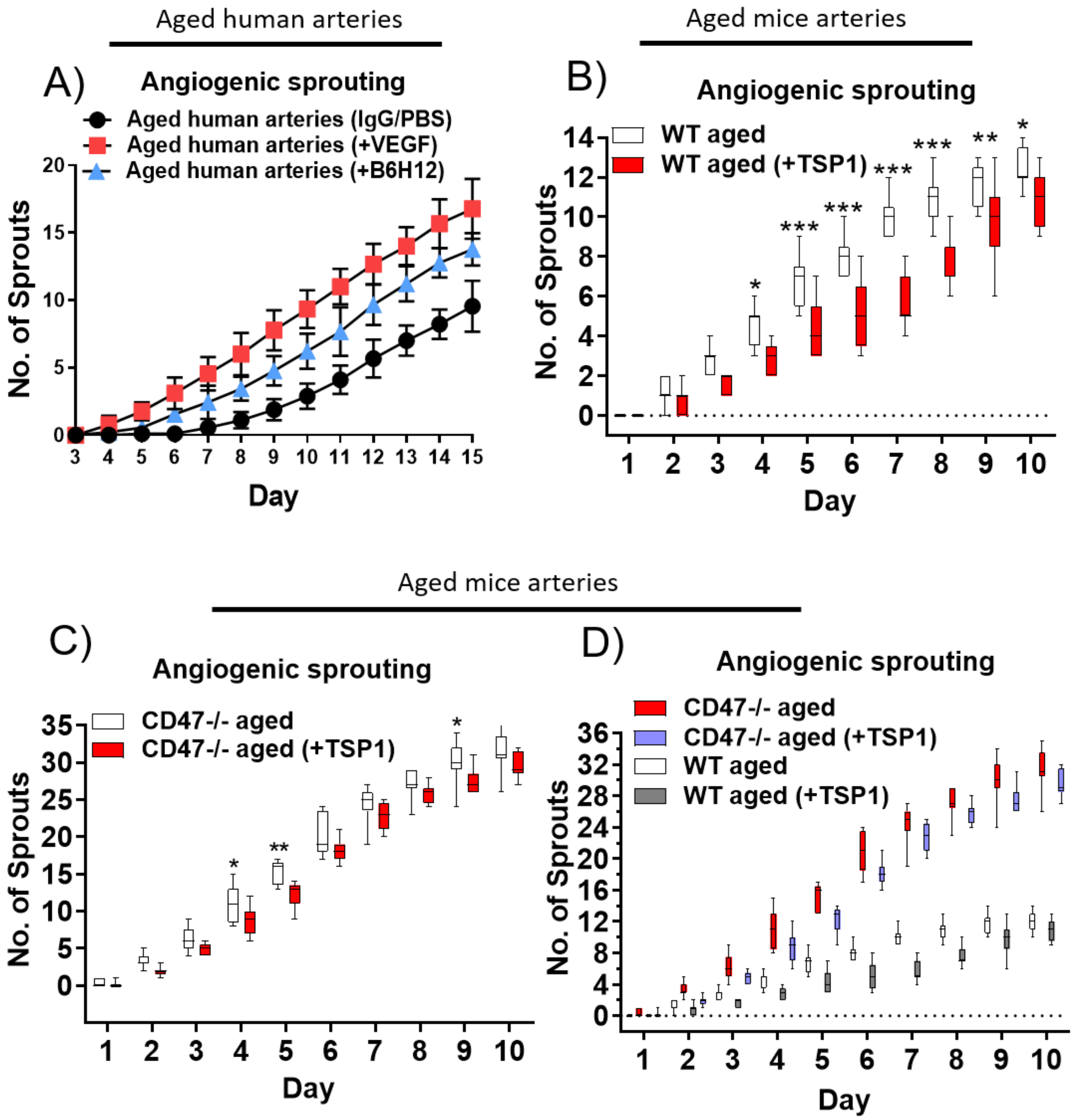

Supplementary Figure 3. Comparison of angiogenic sprouting in human and mouse arteries under various conditions. Side-by-side comparison of angiogenic sprouting in arteries from older individuals treated with a CD47 antibody (2 µg/mL) or VEGF (50ng/mL) (**A**)**.** TSP1 (2.2 nM) inhibited sprouting in aged mice arteries compared to controls (**B**) TSP1 was ineffective to inhibit the increased sprouting seen in aged CD47-null aortic rings (**C**) and side by side comparison of the results (B and C) in **(D**). Side-by-side comparison of results (**C**). Error bars represent the mean ± SEM. 3 wells/vessel, 3 subjects/group. Unpaired t-test between the matched groups of each day. *$p < 0.05$, **$p < 0.01$, ***$p <$ 001.

Supplementary Figure 4

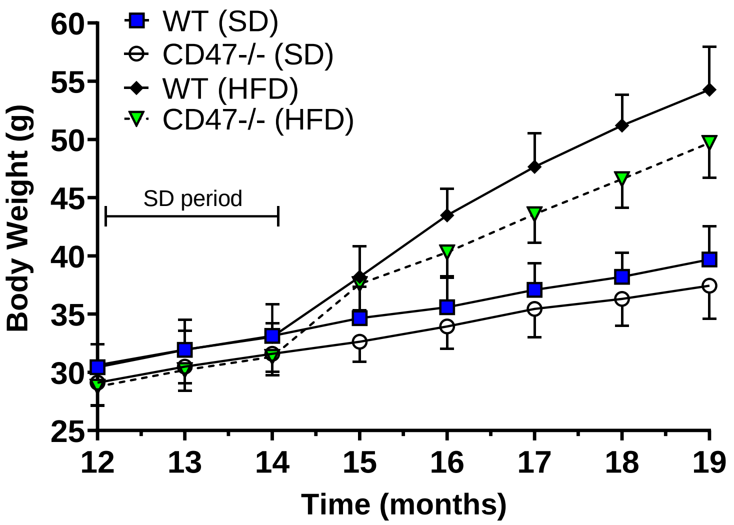

Supplementary Figure 4. Absence of CD47 retards HFD-induced weight gain with age**.** Body weight measurements of wild-type (WT) and CD47-null mice. All groups were on a standard diet (SD) until 14 months of age after which two groups (10 mice/group) were placed on the HFD and weight monitored until 19 months of age. Unpaired t-test between weight measurements of aged WT and CD47-null mice on the HFD, **p* < 0.05, ***p* < 0.01, ****p* < 001.
